# *In-Vitro* Antimicrobial Activities of Grape Seed, Green Tea, and Rosemary Phenolic Extracts Against Liver Abscess Causing Bacterial Pathogens in Cattle

**DOI:** 10.3390/microorganisms12112291

**Published:** 2024-11-11

**Authors:** Harith M. Salih, Raghavendra G. Amachawadi, Qing Kang, Yonghui Li, Tiruvoor G. Nagaraja

**Affiliations:** 1 Department of Clinical Sciences, College of Veterinary Medicine, Kansas State University, Manhattan, KS 66506, USA; hmssalih@vet.ksu.edu; 2 Department of Statistics, College of Arts and Sciences, Kansas State University, Manhattan, KS 66506, USA; kangqing@hotmail.com; 3 Department of Grain Science and Industry, College of Agriculture, Kansas State University, Manhattan, KS 66506, USA; yonghui@ksu.edu; 4 Department of Diagnostic Medicine/Pathobiology, Kansas State University, Manhattan, KS 66506, USA; tnagaraj@vet.ksu.edu

**Keywords:** liver abscesses, feedlot cattle, bacterial pathogens, plant-based phenolic extracts, antibiotic alternatives

## Abstract

Liver abscesses, which occur in finishing cattle, are of significant economic concern to the feedlot industry. The causative agents include both *Fusobacterium necrophorum* subspecies (*F. necrophorum* and *F*. *funduliforme*), *Trueperella pyogenes* (*T. pyogenes*), and *Salmonella enterica* serotype Lubbock (S. Lubbock). Tylosin, a macrolide antibiotic, is supplemented in the feed to reduce liver abscesses. However, due to the concern with emergence of antimicrobial resistance, the antimicrobial activities of the plant-based phenolic compounds could be an antibiotic alternative to control liver abscesses. We investigated the inhibitory activities of phenolic compounds extracted from grape seed, green tea, and rosemary on liver-abscess-causing bacterial pathogens. Total phenolic content was determined spectrophotometrically. Anaerobic Brain–Heart Infusion broth (for *Fusobacterium*) and Muller–Hinton broth (for *S. enterica* and *T. pyogenes*) with phenolic extracts at 0, 0.1, 1, and 2 mg/mL were prepared. Growth was measured at 0, 12, 24 and 48 h by determining bacterial concentrations. A micro-broth dilution method was used to quantify the inhibition. Grape seed and green tea phenolics inhibited growth of both *Fusobacterium* subspecies, *T. pyogenes* and *S. enterica*. Green tea at 1 mg/mL concentration was more effective in inhibiting the growth of *Fusobacterium* when compared to grape seed and rosemary. Green tea at 2 mg/mL was more effective than at 1 mg/mL against *Salmonella*. The inhibitory effect was dose-dependent, which was consistent across all strains within the same bacterial species. The phenolic extracts were inhibitory against *T. pyogenes* with minimum inhibitory concentration ranging from 6.25 to 12.5 µg/mL. Among the phenolic extracts tested, green tea showed the most potent activity, suggesting its strong potential as a natural alternative to conventional antibiotics. Plant-based phenolic compounds supplemented in the feed may have the potential to control liver abscesses.

## 1. Introduction

Liver abscess is a significant economic concern in the feedlot cattle industry, primarily because it is typically detected only at the time of slaughter. The National Beef Quality Audit report found that liver abscesses account for two-thirds of the 20.9% of livers condemned at slaughter [1]. The incidence of liver abscesses in slaughtered cattle in the United States has been observed to range from 0% to 90%, depending on the region, with an average incidence of approximately 20.3% across all U.S. regions [2]. Besides economic losses due to liver condemnations, liver abscesses also impact cattle performance. Consequently, addressing this issue is critical for enhancing both animal welfare and profitability in the cattle industry. Several factors contribute to the incidence of liver abscesses, such as the amount of grain, forage type, grain type, days on feed, breed, gender, season, and geographical location [1]. The primary predisposing factor leading to liver abscess development is chronic ruminal acidosis, which occurs due to the feeding of a high-grain-based finishing diet that is low in roughages. The ruminal acidosis leads to ruminitis, which facilitates bacterial translocation from the rumen to the liver via portal circulation, commonly referred to as the Acidosis–Rumenitis– Liver-Abscess Complex [1]. Liver abscesses develop due to the invasion of bacterial species, which include *Fusobacterium necrophorum* subsp. *necrophorum*, *Fusobacterium necrophorum* subsp. *funduliforme*, *Trueperella pyogenes*, *Bacteroides* spp., *Clostridium* spp., *Pasteurella* spp., *Peptostreptococcus* spp., *Staphylococcus* spp., and *Streptococcus* spp. [3]. More recently, *Salmonella enterica* serotype Lubbock has also been identified in liver abscesses [4]. In feedlot cattle, controlling liver abscesses has traditionally relied on the use of antimicrobial compounds administered in feed, coupled with prudent nutritional management to reduce the risk of ruminal acidosis and subsequent ruminitis. Among the antibiotics used, tylosin is the most common to prevent liver abscesses in feedlot cattle [5]. However, even with tylosin administration, liver abscesses still occur [6], although the rate is significantly lower compared to the cattle not fed tylosin [7]. Despite its effectiveness, tylosin use in cattle feed has come under increased scrutiny, especially since it belongs to the macrolide class, which includes erythromycin, a drug widely used in human medicine. Because of the concern with antimicrobial resistance, the use of tylosin in livestock has been closely monitored under FDA guidelines and the Veterinary Feed Directive implemented in January 2017 [5]. Given these concerns, there is a need for research to explore and evaluate non-antibiotic methods for controlling liver abscesses in cattle. Potential approaches include developing feed formulation that minimize variations in feed intake incorporating specific nutrients and dietary ingredients [8], using feed additives [9,10], and employing probiotics and prebiotics [11]. Another promising area of research is investigating the antimicrobial properties of natural plant-derived phenolic extracts as alternatives to conventional antibiotics. Expanding on this, grape seed, green tea, and rosemary extracts have gained attention for their strong antimicrobial properties, making them promising alternatives to conventional antibiotics [12,13,14].

Grape seeds (*Vitis viniferla* L.) possess substantial antioxidant potential, contributing to the protection against oxidative damage in cells. They have been shown to modulate the expression of antioxidant enzymes, exhibit anti-atherosclerotic and anti-inflammatory effects, and provide protective benefits against certain types of cancer in both animals and humans [15,16]. Furthermore, the antimicrobial properties of grape seed extract can be attributed to its phenolic compounds, particularly resveratrol. Resveratrol induces oxidative damage to bacterial membranes, effectively compromising bacterial viability while sparing host cells. This dual function of grape seeds, acting as both antioxidants and antimicrobials highlights their potential as valuable natural agents in promoting health and preventing disease [17].

Green tea is an unfermented beverage derived from the leaves of the *Camellia sinensis* L. plant, rich in various pharmacologically active components. Tea polyphenols, which encompass approximately 30 different types of compounds, primarily flavonoids, catechins, anthocyanins, and phenolic acids, are particularly noteworthy for their health benefits [18,19]. The antioxidant activity of green tea polyphenols is largely attributed to their unique chemical structure, characterized by the presence of hydroxyl groups and aromatic rings. Additionally, research has demonstrated that green tea exhibits significant antibacterial properties against a variety of pathogens, including *Helicobacter pylori*, as well as major foodborne pathogens, such as *Staphylococcus aureus*, *Escherichia coli*, *Salmonella enterica serovar Typhimurium*, and *Listeria monocytogenes* [20].

Rosemary (*Rosmarinus officinalis* L.) extract is recognized for its diverse bioactive properties, including hepatoprotective, antifungal, antioxidant, insecticidal, and antibacterial effects. These beneficial properties are primarily attributed to the presence of phenolic compounds within the extract [21,22,23]. The broad-spectrum antimicrobial activity is likely due to its soluble bioactive constituents, which may be influenced by the nature of the solvent used for extraction and its polarity. Notably, rosemary extract has demonstrated efficacy against both Gram-positive and Gram-negative bacterial isolates, highlighting its potential as a natural antimicrobial agent [24,25]. Our study is aimed at determining the antibacterial activities of crude phenolic extracts of grape seed, green tea, and rosemary, against pathogens that cause liver abscesses in feedlot cattle.

## 2. Materials and Methods

### 2.1. Plant Extracts

The Phytophenol extracts used in this study include Grape seed extract (*Vitis viniferla* L.), Green tea extract (*Cameillia sinensis O. Ktze*), and Rosemary extract (*Rosmarinus officinalis* L.) (Bulk Supplements: Henderson, NV, USA). The Grape seed extract was standardized to contain 95% Proanthocyanidins.

### 2.2. Extraction of Phytophenols

The maceration method described in the study by Alara et al. [26] was employed to extract the phenolic contents from the plants under investigation. The dried plant powders were further ground, and 100 g of each finely ground plant powder was mixed with 500 mL of 75% acetone solution (Fisher Scientific, Fair Lawn, NJ, USA). The mixture was stirred continuously for 2 h and then stored at −20 °C for 24 h. Afterward, the mixture was centrifuged at 3345 G for 10 min using a cold centrifuge. The supernatant was collected and subjected to acetone removal using a rotary evaporator at 40 °C (Toolots^®^, Colton, CA, USA). The resulting suspension was transferred to a Pyrex container (Corning LLC, Fort Worth, TX, USA) for drying in a freeze dryer, after which the powder was stored at −20 °C until further use.

### 2.3. Measurement of Total Phenolic Contents

The total phenolic content (TPC) was measured following the completion of the extraction process. A 0.1 mL aliquot of the obtained supernatant was thoroughly mixed with 7.9 mL of deionized distilled water and 0.5 mL of Folin–Ciocalteu (Sigma-Aldrich, St. Louis, MO, USA) reagent. After allowing the mixture to sit at room temperature for 5 min, 1.5 mL of Na_2_CO_3_ solution (20%, w/v) was added. The mixture was then left to sit for 2 h. The absorbance was measured at 765 nm using a VWR UV1600-PC spectrophotometer (Randor, PA, USA). Using gallic acid as an external standard, the spectrophotometer results were calculated and expressed as micrograms of gallic acid equivalents (GAEs) per gram of the original supernatant sample (mg GAE/g) [27,28]. The standard curve for the TPC estimation is provided in the Supplementary Figure S1.

### 2.4. Bacterial Strains

Five strains each of *F. necrophorum* subsp. *necrophorum* (2016-13#55, 2016-13#69, 2018-13#23, 2018-13#24, and 2018-13#37) *F. necrophorum* subsp. *funduliforme* (2018-1#6, 2018-1#7, 2018-13#2, 2018-13#16, and 2018-13#35), *Salmonella enterica* serotype Lubbock (2016-13#26, 2016-13#33, 2016-13#34, 2016-13#36, and 2016-13#38), and *Trueperella pyogenes* (2016-13#306, 2016-13#311, 2016-13#315, 2016-13#318, and 2016-13#319) were used in the study. These strains were all isolated from liver abscesses in feedlot cattle [4,29].

### 2.5. Preparation of Bacterial Inoculums

The bacterial species used to prepare the inocula for all experimental treatments included *Fusobacterium necrophorum* subsp. *necrophorum*, *Fusobacterium necrophorum* subsp. *funduliforme*, *Salmonella enterica* serotype Lubbock, and *Trueperella pyogenes* (as stated in the above section). These bacteria were streaked onto blood agar plates (Remel Inn, Lenexa, KS, USA). Both *Fusobacterium* subspecies were incubated anaerobically at 39 °C for 48 h, while *Salmonella enterica* serotype Lubbock was incubated aerobically at 37 °C for 24 h, and *Trueperella pyogenes* was incubated in a CO_2_-enriched environment at 37 °C for 48 h. For both the *Fusobacterium* subspecies, a single colony was taken from the freshly grown plate and suspended in 10 mL of Brain–Heart Infusion (BHI) broth (Becton Dickinson, Sparks, MD, USA) until the turbidity matched the 0.5 McFarland standard. Then, 1 mL of this suspension was transferred into 9 mL of BHI broth. The same method was applied for *Salmonella enterica* serotype Lubbock and *Trueperella pyogenes*, where a single colony was suspended in 10 mL of phosphate-buffered saline (PBS; Becton Dickinson, Sparks, MD, USA) until the turbidity reached the 0.5 McFarland standard, followed by the transfer of 1 mL into 9 mL of Mueller–Hinton (MH) broth (Becton Dickinson, Sparks, MD, USA).

### 2.6. Broth Macro-Dilution Method

To determine the antimicrobial activities of the phenolic extracts, a macro-dilution assay was performed as per the CLSI guidelines with slight modifications [30]. The assays utilized the pre-reduced anaerobically sterilized (PRAS)-BHI broth for both *Fusobacterium* subspecies and Mueller–Hinton (MH) broth for *Salmonella enterica* serotype Lubbock and *Trueperella pyogenes*. Optical density measurements were taken at 24 and 48 h, both with and without phenolic extracts, and cultures were serially diluted ten-fold and appropriate dilutions were plated onto blood agar plates to determine concentrations (CFU per mL). Each run included appropriate controls, such as a bacteria control, DMSO (solvent) control, and phenolic extract controls.

### 2.7. Broth Micro-Dilution Method

The minimum inhibitory concentrations (MICs) of grape seed, green tea, and rosemary phenolic extracts, chlortetracycline (Sigma Aldrich, St. Louis, MO, USA) and tylosin (Sigma Aldrich) were determined using the micro-broth dilution method following the CLSI guidelines [30]. Both chlortetracycline and tylosin antibiotics served as antibiotic controls for comparison with the phenolic extracts. Wells without either antibiotics or phenolic extracts served as negative controls. Antibiotic stock solutions were prepared according to the manufacturer’s instructions to achieve a concentration of 1000 µg/mL, based on their respective potencies. The three phenolic extracts and the antibiotics were tested at concentrations of 100, 50, 25, 12.5, 6.25, 3.125, 1.56, 0.78, 0.39, and 0.195 µg/mL. The assay was performed in 96-well micro titer plates (Becton and Dickinson, Franklin Lakes, NJ, USA). Plates were incubated under anaerobic (*Fusobacterium)*, CO_2_ (*Trueperella*), and aerobic (*Salmonella*) conditions. Results were recorded as either growth or no growth, and the procedure was repeated with different bacterial inocula.

### 2.8. Statistical Analysis

Study employed either a completely randomized design or a randomized incomplete block design, with repeated measurements, to evaluate the effects of each phenolic compound grape seed, green tea, or rosemary at a given concentration against each bacterial species or subspecies. The treatment groups were arranged in a 2-way factorial design, with bacterial strain and growth condition as the factors. The date of the experiment (replications) served as a blocking factor. The growth of bacteria at a given time was measured in optical density (OD) and log10 (CFU/mL). The change in OD from the control represented the absolute difference between control and phenolic extract under the same growth conditions, time, and replication. The fixed effects of the model included strain (which varied among experiments), growth condition (medium + bacteria, medium + DMSO + bacteria, medium + compound + bacteria), time (24 h, 48 h, and, when applicable, 0 and 12 h), as well as all 2-way and 3-way interactions among strain, growth condition, and time. In the randomized complete block design (RCBD) for both grape seed and green tea, the blocking factor also served as a fixed effect. The random effect consisted of the combination of replication, strain, and growth condition. The variance–covariance structure of the random effect was assumed to follow compound symmetry. Additionally, in the RCBD for both grape seed and green tea, the variance–covariance was heterogeneous with respect to growth condition. The effect of growth condition was evaluated at a given time, disregarding the significance of the 3-way interaction. The least-squares means (LSMs) and their standard error of means (SEMs) were employed to evaluate the fixed effects. All tests were two-sided and conducted at the 0.05 significance level, with no multiplicity adjustment applied. Statistical analyses were performed using the Statistical Analysis Software (SAS version 9.4; Cary, NC, USA) through the MIXED procedure, utilizing the DDFM-R option in the MODEL statement.

## 3. Results

### 3.1. Total Phenolic Content

The total phenolic contents of the extracts prepared from grape seed, green tea and rosemary were 614.1 mg, 878.4 mg, and 153.8 mg of gallic acid equivalents (GAE) per g, respectively. These values indicate a substantial variation in phenolic concentration among the different sources, with green tea exhibiting the highest phenolic content, followed by grape seed, and with rosemary showing the lowest.

### 3.2. Broth Macro-Dilution Method

The antibacterial activities of phenolic extracts from grape seed, green tea, and rosemary were evaluated at concentrations of 0.1, 1, and 2 mg/mL against four liver-abscess-causing bacterial pathogens.

#### 3.2.1. Grape Seed Phenolic Extract

The grape seed phenolic extract exhibited significant antibacterial activity against the four liver-abscess-causing pathogens tested. At the lowest concentration of 0.1 mg/mL, the extract completely inhibited the growth of *T. pyogenes* (*p* < 0.0001). At 1 mg/mL concentration, there was complete inhibition of *T. pyogenes* (*p* < 0.0001), partial inhibition of both subspecies of *F. necrophorum* (*necrophorum* and *funduliforme*) (*p* < 0.0001), and no effect on *Salmonella enterica* serotype Lubbock (*p* > 0.05). At the highest concentration of 2 mg/mL, the grape seed extract achieved complete growth inhibition of both subspecies of *F. necrophorum* (*necrophorum* and *funduliforme*) (*p* < 0.0001), but had no effect on *S. enterica* serotype Lubbock (*p* > 0.05) (Table 1).

#### 3.2.2. Green Tea Phenolic Extract

The green tea phenolic extract demonstrated potent antibacterial activity across all concentrations tested. At 0.1 mg/mL, the extract completely inhibited the growth of *T. pyogenes* and both subspecies of *F. necrophorum* (*necrophorum* and *funduliforme*) (*p* < 0.0001), although *S. enterica* serotype Lubbock remained unaffected (*p* > 0.05). Increasing the concentration to 1 mg/mL resulted in significant growth inhibition of all four bacterial species, including *S. enterica* serotype Lubbock (*p* < 0.001). Notably, a significant 3-way interaction (bacteria, growth condition, and time) was observed for the green tea extract at 0.1 mg/mL, specifically against *F. necrophorum* subsp. *necrophorum* (*p* = 0.0001) and *F. necrophorum* subsp. *funduliforme* (*p* < 0.001) (Table 1).

#### 3.2.3. Rosemary Phenolic Extract

The rosemary phenolic extract also showed strong inhibitory effects, particularly at higher concentrations. At 0.1 mg/mL, it completely inhibited the growth of *T. pyogenes* (*p* < 0.0001). At 1 mg/mL, the rosemary extract exhibited a broader range of inhibitory effects, including complete inhibition of *T. pyogenes* (*p* < 0.0001) and partial inhibition of both subspecies of *F. necrophorum* (*necrophorum* and *funduliforme*), and *S. enterica* serotype Lubbock (*p* < 0.001). A significant 3-way interaction was observed for the rosemary extract at 0.1 mg/mL against *F. necrophorum* subsp. *necrophorum* (*p* = 0.0002), and at 2 mg/mL, the extract significantly inhibited both *F. necrophorum* subsp. *necrophorum* (*p* = 0.0092) and *F. necrophorum* subsp. *funduliforme* (*p* = 0.0110) (Table 1).

The impact of grape seed, green tea, and rosemary phenolic extracts on the bacterial counts of liver-abscess-causing pathogens was evaluated after 24 and 48 h of incubation (Table 2). For *F. necrophorum* subsp. *necrophorum* treated with grape seed extract at 1 mg/mL, significant effects were observed for strain and condition (*p* < 0.0001), while interactions with time were not significant (*p* > 0.05). Similarly, green tea extract showed significant results for strain and growth condition on *F. necrophorum* subsp. *necrophorum* (*p* = 0.0004) without significant interactions involving time (*p* > 0.05). In contrast, rosemary extract produced significant effects for strain and growth condition on *F. necrophorum* subsp. *necrophorum* (*p* < 0.0001), though interactions with time and condition were not significant (*p* > 0.05). For *F. necrophorum* subsp. *funduliforme*, grape seed extract at 1 mg/mL resulted in significant effects for strain and condition, and their interactions with time (*p* < 0.0001). Rosemary extract also produced significant main effects and interactions for strain, condition, and time (*p* < 0.0001), although some interactions were not significant (*p* > 0.05).

The effects of grape seed, green tea, and rosemary phenolic extracts on the bacterial concentrations of liver-abscess-causing pathogens were assessed at 24 and 48 h of incubation (Table 3). For *F. necrophorum* subsp. *necrophorum*, treatment with grape seed extract at 1 mg/mL significantly reduced bacterial counts compared to both the bacteria control and DMSO-treated groups at 24 h (LSM = 4.78, SEM = 0.15) and 48 h (LSM = 4.90, SEM = 0.15), with the controls showing much higher values (LSM = 9.62–9.72, SEM = 0.21). Similarly, *F. necrophorum* subsp. *funduliforme* treated with grape seed extract exhibited significantly lower bacterial counts at both 24 h (LSM = 4.61, SEM = 0.09) and 48 h (LSM = 4.99, SEM = 0.09), compared to controls. Green tea extract also demonstrated significant inhibitory effects on *F. necrophorum* subsp. *necrophorum*, reducing bacterial concentrations to 1.23 (SEM = 0.22) and 1.75 (SEM = 0.22) at 24 and 48 h, respectively, compared to the control groups (LSM = 7.22–9.15, SEM = 0.64–0.68). Lastly, rosemary extract showed substantial inhibition of both subspecies of *F. necrophorum* (*necrophorum* and *funduliforme*) at 24 and 48 h, with LSM values ranging from 2.70 to 4.60 (SEM = 0.27–0.30), compared to the control groups (LSM = 8.27–10.87, SEM = 0.38–0.43). These results indicate the potential of these phenolic extracts, particularly grape seed, green tea, and rosemary, in reducing the bacterial load of liver-abscess-causing pathogens.

### 3.3. Broth Micro-Dilution Method

**Figure 1:**
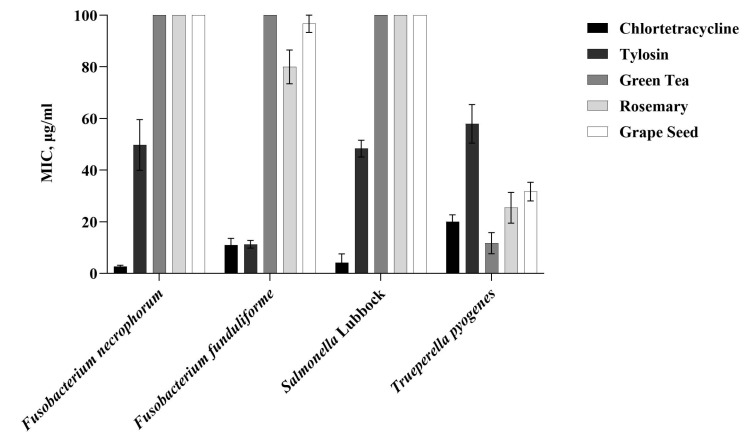
Minimum inhibitory concentrations (MICs) of antibiotics (chlortetracycline and tylosin) and phenolic extracts (green tea, rosemary, grape seed) against liver-abscess-causing bacterial pathogens.

The susceptibility of the liver-abscess-causing pathogens to grape seed, green tea, and rosemary phenolic extracts was further evaluated by determining the minimum inhibitory concentration (MIC). The isolates of *F. necrophorum* subsp. *necrophorum*, *F. necrophorum* subsp. *funduliforme*, and *Salmonella enterica* serotype Lubbock demonstrated resistance to these phenolic extracts at 1 mg/mL concentrations, with MIC values exceeding 100 µg/mL. In contrast, *Trueperella pyogenes* showed susceptibility to all three phenolic extracts, with MIC values of 31.67 µg/mL for grape seed, 11.67 µg/mL for green tea, and 25.42 µg/mL for rosemary. These values were compared to the MICs of the antibiotics, chlortetracycline and tylosin, which were 20 µg/mL and 57.92 µg/mL, respectively. Additionally, *F. necrophorum* subsp. *necrophorum* isolates had MIC values of 2.66 µg/mL for chlortetracycline and 49.7 µg/mL for tylosin, while *F. necrophorum* subsp. *funduliforme* isolates exhibited MIC values of 11 µg/mL for chlortetracycline and 11.25 µg/mL for tylosin. The *Salmonella enterica* serotype Lubbock isolates had MIC values of 4.2 µg/mL for chlortetracycline and 48.33 µg/mL for tylosin (Figure 1).

## 4. Discussion

Liver abscesses are the primary liver abnormality observed in feedlot cattle and typically go undetected until animals are processed at slaughter plants [7]. These abscesses are the consequence of ruminal acidosis and rumenitis, which are conditions that develop when cattle are fed diets high in readily fermentable carbohydrates, particularly when these diets are low in roughage. The resulting decrease in ruminal pH leads to inflammation and damage to the epithelial cell layer of the rumen, allowing liver-abscess-causing pathogens to enter the bloodstream and ultimately form abscesses in the liver [24]. Due to multiple factors contributing to liver abscess formation, this condition is often referred to as the “acidosis–rumenitis–liver-abscess complex”. Studies have identified *F. necrophorum* subsp. *necrophorum* as the primary causative agent, with *T. pyogenes* acting as a secondary pathogen [1]. Additionally, recent research has identified the presence of *Salmonella enterica* isolated from liver abscesses in cattle, marking the first report of this pathogen in such cases [4]. Several methods have been employed to control liver abscesses in cattle, with the use of antimicrobial compounds, such as tylosin, chlortetracycline, and virginiamycin, being the most common [1,3,8]. Although studies have demonstrated the effectiveness of these antibiotics in mitigating liver abscesses, there are growing concerns about the use of antibiotics in the feedlot industry due to the potential development of antibiotic resistance. This underscores the need for research into alternative strategies that can replicate the effects of these antibiotics without contributing to resistance [25]. Antimicrobial resistance (AMR) is a significant global challenge, requiring action to prevent the spread of multi-drug-resistant (MDR) pathogens [31,32]. The indiscriminate use of antimicrobials has driven AMR pathogens, leading to the exhaustion of antibiotics [33,34]. Developing new generations of antibiotics is increasingly difficult due to financial, technical, and resistance-related challenges [35]. Consequently, there is a growing interest in alternative approaches, such as the use of phytochemicals, to reduce reliance on antibiotics [36,37]. Secondary metabolites in plants, known as phenolic compounds, are recognized for their bioactive properties, including their ability to eliminate free radicals and chelate metals. These compounds are important natural molecules that contribute to the plant’s defense mechanisms, adaptation, and pigmentation processes [38]. Additionally, phenolic compounds play a significant role in preventing certain human diseases, such as cardiovascular conditions, diabetes, and cancer [39]. While profiling can certainly provide insights into composition–activity relationships, we believe that our approach of using well-documented extracts aligns more closely with the practical needs of the industry. Our goal was to evaluate their potential in a feedlot setting rather than conduct a comprehensive chemical analysis, keeping the research focused on application-driven outcomes that will directly contribute to improving cattle health and feeding efficiency.

Among plant-derived phenolic extracts, grape seed, green tea, and rosemary are noteworthy. Grape seeds, a by-product of the agro-industrial sector, can pose socioeconomic and environmental challenges if not properly managed [40]. Studies have demonstrated that the phenolic content extracted from different varieties of grape seeds possess significant antimicrobial and antioxidant capacities. For instance, polyphenols extracted from the seeds and skins of red grapes have shown antimicrobial activity against *Staphylococcus aureus*, *Bacillus cereus*, and *Escherichia coli*, as evidenced by agar diffusion method and minimum inhibitory concentration (MIC) assays [41]. However, the effectiveness of grape seed phenolic extracts can vary depending on the microorganism. For example, one study found that these extracts were ineffective against certain fungi, including *Botrytis cinerea*, *Penicillium expansum*, *Aspergillus niger*, and *Alternaria alternata*, but they were effective against bacterial strains like *Staphylococcus aureus* and *Streptococcus pneumoniae* [42]. Another study highlighted the efficacy of grape seed phenolic extracts in suppressing the growth of food-related pathogens, such as *Listeria monocytogenes*, *Pseudomonas aeruginosa*, *Salmonella Typhimurium*, *Bacillus* spp., and *Campylobacter* spp. [43]. The antimicrobial activity of grape seed extracts is largely attributed to the presence of stigmasterol, a sterol molecule that can induce surface interactions and pore formation in bacterial cell walls, leading to the degradation of bacterial components. Additionally, the presence of tannins in grape seeds may contribute to their antimicrobial properties by inactivating various microbial activities, including enzymes, cell-envelope transport proteins, and microbial adhesions, as well as by modifying the morphology of microorganisms and forming complexes with polysaccharides [42].

Studies on green tea have demonstrated that its polyphenols exhibit numerous biological properties and health benefits. In a comparative study evaluating the antibacterial activities of four tea varieties, including green tea, all varieties were effective against the tested bacterial strains. Notably, Gram-positive bacteria such as *Staphylococcus aureus* and *Enterococcus faecalis* were more susceptible to the tea extracts than Gram-negative bacteria, like *Salmonella Typhimurium* and *Escherichia coli*. Among the varieties tested, green tea exhibited the most potent antibacterial activity, as determined by the minimum inhibitory concentration (MIC) test [44]. However, the application of green tea extracts in the food industry is limited by their sensitivity to high temperature, oxygen, and pH [45]. The catechin polyphenols in green tea are particularly prone to degradation or epimerization when exposed to these factors, leading to a reduction in their efficacy [46]. Furthermore, when green tea phenolic extracts are dried, they become hygroscopic and sticky due to the presence of sugars, resulting in an unstable shelf life, as they readily absorb moisture and degrade [47].

Rosemary, an aromatic evergreen shrub belonging to the *Lamiaceae* family, is widely distributed and has been extensively studied for its phytophenol extracts [48]. The polyphenolic extracts of rosemary contain major components, such as carnosic acid, rosmarinic acid, carnosol, and hesperidin. Among these, carnosic acid, carnosol, and cyclic diterpene diphenols are the most effective constituents of rosemary’s antioxidants. Additional components include epirosmanol, rosmanol, isorosmanol, and methylcarnosate [49]. The inhibitory effect of rosemary’s phenolic extracts is primarily attributed to their interaction with bacterial cell membranes, which occurs through various mechanisms, such as leakage of cellular components, alterations in genetic material and nutrient production, changes in fatty acid synthesis, and disruption of electron transport. These interactions also affect membrane proteins, leading to a loss of membrane structure and functionality [20,50,51]. In a study investigating the antibacterial activity of rosemary phenolic extracts against multidrug-resistant clinical isolates and meat-borne pathogens, the ethanol phenolic extract of rosemary was found to inhibit the growth of both Gram-positive and Gram-negative isolates. Among the Gram-positive isolates, *Staphylococcus aureus* and *Enterococcus* sp. exhibited high-to-moderate inhibition, while *Streptococcus pyogenes* showed lower inhibition levels. For the Gram-negative isolates, the highest inhibition was observed against *Salmonella* sp., with moderate inhibition against *Shigella* sp., *Klebsiella pneumoniae*, *Pseudomonas aeruginosa*, and *E. coli*. The lowest levels of inhibition were noted against *Proteus* sp. and *Campylobacter* sp. [52].

The current study aimed to evaluate the antibacterial effects of phenolic extracts from grape seed, green tea, and rosemary against liver-abscess-causing pathogens, specifically. *F. necrophorum* subsp. *necrophorum*, *F. necrophorum* subsp. *funduliforme*, *Salmonella enterica* serotype Lubbock, and *Trueperella pyogenes*. The results indicated that both subspecies of *F. necrophorum* (*necrophorum* and *funduliforme*), and *Salmonella enterica* serotype Lubbock were resistant to grape seed, green tea, and rosemary phenolic extracts at 1 mg/mL concentrations. In contrast, *T. pyogenes* was highly susceptible to all three phenolic extracts. The significantly lower MIC values of green tea and grape seed extracts suggest their strong potential as natural antibacterial agents against *T. pyogenes*. Interestingly, both subspecies of *F. necrophorum* (*necrophorum* and *funduliforme*) showed different responses to standard antibiotics, chlortetracycline and tylosin. These differences highlight the variable susceptibility of these pathogens to antibiotics and suggest a need for alternative treatment strategies, especially in cases where resistance is evident. Furthermore, the time-course analysis demonstrated that grape seed, green tea, and rosemary phenolic extracts significantly reduced bacterial concentrations of both subspecies of *F. necrophorum* (*necrophorum* and *funduliforme*) at both 24 and 48 h of incubation. Notably, green tea exhibited the strongest inhibitory effect, reducing *F. necrophorum* subsp. *necrophorum* concentrations to as low as 1.23 µg/mL after 24 h, which was sustained at 1.75 µg/mL after 48 h. Similarly, grape seed and rosemary extracts effectively decreased bacterial counts, with rosemary showing significant reductions in *F. necrophorum* subsp. *funduliforme* concentrations at both time points. The findings suggest that the phenolic extracts, particularly green tea, could serve as promising alternatives to traditional antibiotics in reducing liver-abscess-causing pathogens. The varying efficacy of these extracts across different bacterial species emphasizes the importance of understanding pathogen-specific responses to treatments. The significant reduction in bacterial counts at both 24 and 48 h indicates the potential for these extracts to be integrated into livestock management practices, particularly for the prevention and control of liver abscesses in cattle. The findings revealed that these phenolic extracts exhibited varying degrees of antimicrobial activity, with notable differences in their efficacy depending on the bacterial species, extract type, and concentration. However, while the study demonstrates the potential of these natural extracts, further research is necessary to understand the underlying mechanisms of their antibacterial activity, in vivo efficacy, and overall safety of the extracts. Building on our application-driven approach, future studies will explore the integration of these crude extracts into practical feedlot management systems and investigate their impact on antimicrobial resistance, along with feed efficiency and animal welfare. Chemical profiling, combined with in vivo studies, would help us to identify the active ingredients present in these crude phenolic extracts and better understand their mode of action. This could pave the way for securing funding to pursue a larger full-scale longitudinal feeding trial in feedlot cattle aimed at full mechanistic validation of these compounds. Investigations into the synergistic effects of these phenolic compounds with existing antibiotics could also offer valuable insights into developing more effective treatment strategies that can be used in livestock health management.

## 5. Conclusions

In conclusion, this study highlights the significant antibacterial properties of grape seed, green tea, and rosemary phenolic extracts against key liver-abscess-causing bacterial pathogens in cattle. Among the extracts tested, green tea demonstrated the most potent activity, suggesting its strong potential as a natural alternative to conventional antibiotics. The differential efficacy observed across bacterial species underscores the importance of tailored treatment strategies in managing liver abscesses and suggests that these natural compounds could play a valuable role in reducing the reliance on traditional antibiotics. These findings contribute to the growing body of research supporting the use of natural compounds as alternatives to antibiotics in livestock, which is critical in the ongoing effort to mitigate antimicrobial resistance in animal agriculture. Such investigations could pave the way for more sustainable and effective approaches to livestock health management, ultimately contributing to improved animal welfare and food safety.

## Figures and Tables

**Table 1:** The effects of grape seed, green tea, and rosemary phenolic extracts on the growth, measured by optical density, of liver-abscess-causing pathogens at 0, 12, 24, and 48 h of incubation.

Bacteria	Phenolic Extracts	Concentration Tested (mg/mL)	*p*-Values							
			Replication	Strain	Growth Condition	Strain * Growth Condition (2-Way Interaction)	Time	Strain * Time (2-Way Interaction)	Growth Condition * Time (2-Way Interaction)	Strain * Growth Condition * Time (3-Way Interaction)
*Fusobacterium necrophorum* subsp. *necrophorum*	Grape seed	1	-	0.9928	0.0003	0.9992	<0.0001	0.8995	<0.0001	0.9909
*Fusobacterium necrophorum* subsp. *funduliforme*	Grape seed	1	-	0.9143	0.0023	0.8008	<0.0001	0.3004	<0.0001	0.5370
*Fusobacterium necrophorum* subsp. *necrophorum*	Grape seed	2	0.0201	0.9754	0.0022	0.6221	<0.0001	0.1228	<0.0001	0.6699
*Fusobacterium necrophorum* subsp. *funduliforme*	Grape seed	2	0.0176	0.6959	0.0051	0.5194	<0.0001	0.4498	<0.0001	0.4845
*Fusobacterium necrophorum* subsp. *necrophorum*	Green tea	0.1	-	0.1063	<0.0001	0.0169	<0.0001	0.0009	<0.0001	0.0001
*Fusobacterium necrophorum* subsp. *funduliforme*	Green tea	0.1	-	0.0042	<0.0001	0.0024	<0.0001	<.0001	<0.0001	<0.0001
*Fusobacterium necrophorum* subsp. *necrophorum*	Green tea	1	0.9151	0.8256	0.0374	0.9311	<0.0001	0.0162	<0.0001	0.1001
*Fusobacterium necrophorum* subsp. *funduliforme*	Green tea	1	0.5745	0.6105	0.0309	0.7263	<0.0001	0.6430	<0.0001	0.4465
*Salmonella enterica* serotype Lubbock	Green tea	1	-	0.0089	<0.0001	0.3375	<0.0001	0.4992	<0.0001	0.1494
*Salmonella enterica* serotype Lubbock	Green tea	2	-	0.2230	<0.0001	0.6609	<0.0001	0.9963	<0.0001	0.9998
*Fusobacterium necrophorum* subsp. *necrophorum*	Rosemary	1	-	0.4324	<0.0001	0.5407	<0.0001	0.0997	<0.0001	0.0002
*Fusobacterium necrophorum* subsp. *funduliforme*	Rosemary	1	-	0.2968	<0.0001	0.1652	<0.0001	0.0982	<0.0001	0.1070
*Salmonella enterica* serotype Lubbock	Rosemary	1	-	0.9990	0.0001	1.0000	<0.0001	1.0000	<0.0001	1.0000
*Fusobacterium necrophorum* subsp. *necrophorum*	Rosemary	2	-	0.0143	<0.0001	0.0551	<0.0001	0.3012	<0.0001	0.0092
*Fusobacterium necrophorum* subsp. *funduliforme*	Rosemary	2	-	0.0082	<0.0001	0.0014	<0.0001	0.0268	<0.0001	0.0110
Alpha = 0.05										

* Growth condition = bacteriological culture medium alone or in combination with bacteria; bacteriological culture medium alone or in combination with DMSO (Dimethyl sulfoxide); bacteriological culture medium alone or in combination with phenolic extract compounds.

**Table 2:** The effects of grape seed, green tea, and rosemary phenolic extracts on the concentrations of liver-abscess-causing pathogens at 24 and 48 h of incubation.

Bacteria	Phenolic Extracts	Concentration (mg/mL)	*p*-values							
			Replication	Strain	Growth Condition	Strain * Growth Condition (2-Way Interaction)	Time	Strain * Time (2-Way Interaction)	Growth Condition * Time (2-Way Interaction)	Strain * Growth Condition * Time (3-Way Interaction)
*Fusobacterium necrophorum* subsp. *necrophorum*	Grape seed	1	-	0.3603	<0.0001	0.2315	0.4216	0.3093	0.1574	0.4378
*Fusobacterium necrophorum* subsp. *funduliforme*	Grape seed	1	-	0.0131	<0.0001	0.0109	<0.0001	0.0002	<0.0001	0.0002
*Fusobacterium necrophorum* subsp. *necrophorum*	Green tea	1	0.4278	0.8984	0.0004	0.8158	0.7739	0.5887	0.8503	0.9274
*Fusobacterium necrophorum* subsp. *necrophorum*	Rosemary	1	-	0.5622	<0.0001	0.3149	0.1592	0.9817	0.2339	0.9812
*Fusobacterium necrophorum* subsp. *funduliforme*	Rosemary	1	-	0.3531	<0.0001	0.5415	0.0021	0.1215	0.0063	0.1021
Alpha = 0.05										

* Growth condition = bacteriological culture medium alone or in combination with bacteria; bacteriological culture medium alone or in combination with DMSO (Dimethyl sulfoxide); bacteriological culture medium alone or in combination with phenolic extract compounds.

**Table 3:** The effects of grape seed, green tea, and rosemary phenolic extracts on the bacterial concentrations (log) of liver-abscess-causing pathogens at 24 and 48 h of incubation.

Bacteria	Phenolic Extracts	Concentration Tested (mg/mL)			Bacterial Concentration (Log Values)		
			Time	Growth Condition	LSM	SEM	*
*Fusobacterium necrophorum* subsp. *necrophorum*	Grape seed	1	24 h	Bacteria control	9.62	0.21	A
				DMSO + Bacteria	9.72	0.21	A
				Phenolic extract + Bacteria	4.78	0.15	B
			48 h	Bacteria control	9.50	0.21	A
				DMSO + Bacteria	9.57	0.21	A
				Phenolic extract + Bacteria	4.90	0.15	B
*Fusobacterium necrophorum* subsp. *funduliforme*	Grape seed	1	24 h	Bacteria control	9.91	0.13	A
				DMSO + Bacteria	9.11	0.13	B
				Phenolic extract + Bacteria	4.61	0.09	C
			48 h	Bacteria control	9.97	0.13	A
				DMSO + Bacteria	5.82	0.13	B
				Phenolic extract + Bacteria	4.99	0.09	C
*Fusobacterium necrophorum* subsp. *necrophorum*	Green tea	1	24 h	Bacteria control	8.12	0.68	A
				DMSO + Bacteria	9.15	0.64	A
				Phenolic extract + Bacteria	1.23	0.22	B
			48 h	Bacteria control	7.22	0.68	A
				DMSO + Bacteria	9.10	0.64	A
				Phenolic extract + Bacteria	1.75	0.22	B
*Fusobacterium necrophorum* subsp. *necrophorum*	Rosemary	1	24 h	Bacteria control	10.72	0.43	A
				DMSO + Bacteria	7.31	0.43	B
				Phenolic extract + Bacteria	3.51	0.30	C
			48 h	Bacteria control	10.87	0.43	A
				DMSO + Bacteria	7.37	0.43	B
				Phenolic extract + Bacteria	4.60	0.30	C
*Fusobacterium necrophorum* subsp. *funduliforme*	Rosemary	1	24 h	Bacteria control	10.40	0.38	A
				DMSO + Bacteria	8.59	0.38	B
				Phenolic extract + Bacteria	2.81	0.27	C
			48 h	Bacteria control	8.75	0.38	A
				DMSO + Bacteria	8.27	0.38	A
				Phenolic extract + Bacteria	2.70	0.27	B
Alpha = 0.005							

* Growth condition = bacteria control—bacteriological culture medium and bacteria; DMSO control—bacteriological culture medium and DMSO (Dimethyl sulfoxide); phenolic extract and bacteria—bacteriological culture medium with bacteria and phenolic extract.

## Data Availability

Data are contained within the article.

## Supplementary Materials

The following supporting information can be downloaded at: https://www.mdpi.com/article/10.3390/microorganisms12112291/s1. Supplementary Figure S1: Gallic acid standard curve for total phenolic content (TPC) estimation.
