# Supplementary material for: In-Vitro Antimicrobial Activities of Grape Seed, Green Tea, and Rosemary Phenolic Extracts Against Liver Abscess Causing Bacterial Pathogens in Cattle
Source: Microorganisms. 2024 Nov 11;12(11):2291. doi: 10.3390/microorganisms12112291 (PMC11596820; doi:10.3390/microorganisms12112291)
Supplement: Supplementary file 1 [file microorganisms-12-02291-s001.zip › microorganisms-3317719-supplementary.pdf]

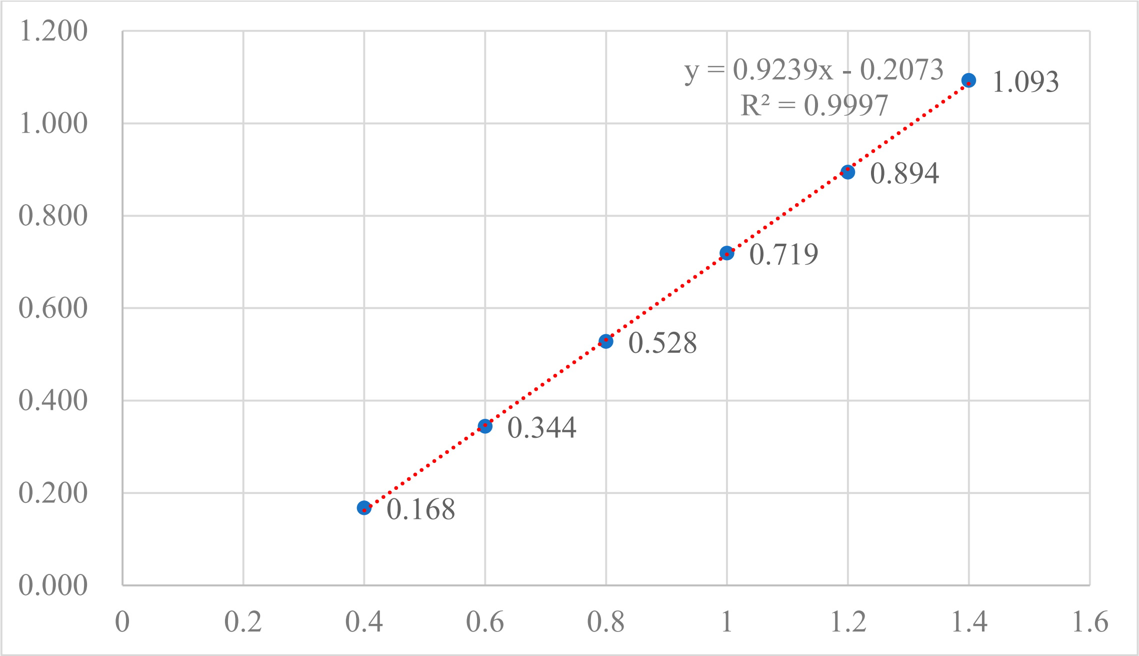

Supplementary Figure S1: Gallic acid standard curve for total phenolic content (TPC) estimation. The standard curve was generated using known concentrations of gallic acid (X-axis). The linear regression equation is y=0.9239x−0.2073y = 0.9239x - 0.2073y=0.9239x−0.2073, with an R2R^2R2 value of 0.9997, indicating a high degree of fit between the concentration and absorbance. This curve was used to estimate the phenolic content of unknown samples based on their absorbance values. Each point represents the mean absorbance of duplicate measurements at varying concentrations of gallic acid.
